# The Role of Expectancy Violation in Extinction Learning: A Two-Day Online Fear Conditioning Study

**DOI:** 10.32872/cpe.9627

**Published:** 2023-06-29

**Authors:** Daniel Gromer, Lea K. Hildebrandt, Yannik Stegmann

**Affiliations:** 1 Department of Psychology, University of Würzburg, Würzburg, Germany; Philipps-University of Marburg, Marburg, Germany

**Keywords:** fear, anxiety, exposure therapy, inhibitory learning, expectancy violation, fear conditioning

## Abstract

**Background:**

Exposure therapy is at the core of the treatment of pathological anxiety. While the inhibitory learning model proposes a framework for the mechanisms underlying exposure therapy, in particular expectancy violation, causal evidence for its assumptions remains elusive. Therefore, the aim of the current study was to provide evidence for the influence of expectancy violation on extinction retention by manipulating the magnitude of expectancy violation during extinction learning.

**Method:**

In total, 101 individuals completed a web-based fear conditioning protocol, consisting of a fear acquisition and extinction phase, as well as a spontaneous recovery and fear reinstatement test 24h later. To experimentally manipulate expectancy violation, participants were presented only with states of the conditioned stimulus that either weakly or strongly predicted the aversive outcome. Consequently, the absence of any aversive outcomes in the extinction phase resulted in low or high expectancy violation, respectively.

**Results:**

We found successful fear acquisition and manipulation of expectancy violation, which was associated with reduced threat ratings for the high compared to the low expectancy violation group directly after extinction learning. On Day 2, inhibitory CS-noUS associations could be retrieved for expectancy ratings, whereas there were no substantial group differences for threat ratings.

**Conclusion:**

These findings indicate that the magnitude of expectancy violation is related to the retrieval of conscious threat expectancies, but it is unclear how these changes translate to affective components (i.e., threat ratings) of the fear response and to symptoms of pathological anxiety.

## Highlights

- Causal evidence for expectancy violation as a key mechanism of exposure therapy is sparse.
- The current study experimentally manipulates the magnitude of expectancy violation.
- High expectancy violation promotes extinction retention for threat expectancy ratings.
- Affective components of the fear response were not affected by expectancy violation.

Exposure therapy is considered the gold standard for the treatment of a variety of mental disorders, particularly anxiety disorders (Hofmann & Smits, 2008; Norton & Price, 2007). Exposure-based interventions focus on repeated confrontations with the fearful object or situation, which typically results in fear extinction characterized as the reduction in fear responses (e.g., behavioral avoidance, physiological arousal, subjective feelings of fear) over time. There is unanimous evidence for the effectiveness of exposure therapy for the treatment of anxiety disorders (Butler et al., 2006; Carpenter et al., 2018; Hofmann & Smits, 2008; Norton & Price, 2007). Yet, there is a considerable amount of patients, who do not profit from treatment, which is reflected in high rates of nonresponding and relapse (Ali et al., 2017; Arch & Craske, 2009, 2011; Taylor et al., 2012). The main obstacle to increasing the effectiveness of exposure-based interventions is that the underlying mechanisms are not yet fully understood (Cooper et al., 2017; Craske et al., 2008; Craske et al., 2014).

**Figure 1:**
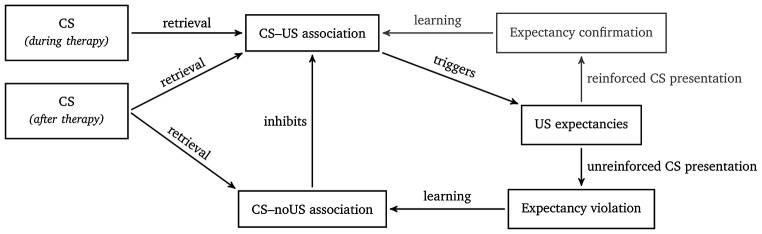
Overview of the Inhibitory Learning Model *Note.* The exposure to a conditioned stimulus (CS, e.g., a dog), associated with an unconditioned stimulus (CS-US association, e.g., getting bitten), triggers the expectation of an aversive outcome (US-expectancy, e.g., getting bitten again). During therapy, patients are exposed to the CS, while the expected aversive outcome is omitted (expectancy violation, e.g., the patient was not attacked by the dog), giving rise to a new CS-noUS memory trace, which is able to inhibit the original CS-US association.

The inhibitory learning model suggests extinction learning as a key mechanism underlying exposure-based interventions resulting from a discrepancy between the conscious expectancy of an aversive event and its omission (Craske et al., 2014; Craske et al., 2022; Rescorla & Wagner, 1972). Instead of erasing the original stimulus-harm association, the omission of the expected aversive outcome (expectancy violation) is assumed to generate a new associative memory trace between the stimulus and the absence of harm, which is thought to exert an inhibitory influence on the original stimulus-harm association (Bouton, 1993; Bouton & King, 1983; Quirk & Mueller, 2008). See Figure 1 for a graphical summary of the processes underlying the inhibitory learning model. To take advantage of inhibitory learning and expectancy violation during therapy, patients should become aware of their expectations for the upcoming exposure session and focus on the discrepancy between the expected and the actual outcome during exposure. In summary, the inhibitory learning model predicts that the strength of expectancy violation is positively related to symptom reduction and thus to the outcome of exposure therapy (Craske et al., 2014; Craske et al., 2022).

Until now, although the inhibitory learning model provides a plausible mechanistic explanation for extinction, studies demonstrating unanimous evidence in support of the role of expectancy violation for positive treatment outcomes are sparse (Craske et al., 2022). While recent models provide a comprehensive framework for studying the mechanisms underlying expectancy violation (Panitz et al., 2021), more research is needed that specifically tests the key mechanisms of the inhibitory learning model. To address this issue, Pavlovian fear conditioning protocols are well suited to examine changes in threat expectancy and thus allow to experimentally test the prediction of the inhibitory learning model that expectancy violation leads to enhanced fear extinction. In fear conditioning paradigms, one conditioned stimulus (CS+) is repeatedly paired with an aversive event (US), resulting in a CS-US association (Pavlov, 1927). During the following extinction phase, US delivery is usually omitted to generate a second CS-noUS association. At a later timepoint, the spontaneous recovery of the CS-US and CS-noUS associations can be tested by re-presenting the CS, while reinstatement of conditioned fear is usually tested by repeating the CS after an US presentation. Using fear conditioning paradigms, extinction learning has been associated with the activation of inhibitory circuits including the ventromedial prefrontal cortex (vmPFC), potentially reflecting the neural correlate of the inhibitory influence of the CS-noUS association on the original CS-US association (Milad & Quirk, 2012). However, how the extent of expectancy violation relates to the inhibitory influence of the CS-noUS association is less well understood. For example, Brown et al. (2017) investigated the relationship between expectancy violation and extinction retention, i.e., the persistent extinction at a follow-up reinstatement test. The authors demonstrated that the variation in US-expectancy during extinction learning, rather than the decline in subjective or psychophysiological fear responding, predicted extinction retention at a follow-up test. These results provide correlational evidence for the role of expectancy violation in extinction learning. Importantly, variation in US-expectancy during extinction as an index for expectancy violation only predicted US-expectancy ratings but not subjective fear or facial EMG at the reinstatement test. In another fear conditioning study by Scheveneels, Boddez, Vervliet, et al. (2019) a hierarchical extinction (i.e., presenting stimuli that increasingly signal the US with an incrementally increasing probability) was compared to a random extinction. Although random extinction led to more expectancy violation during extinction, this did not result in improved CS-discrimination at a follow-up test. However, across groups, the amount of expectancy violation and the variability in US-expectancy during extinction were both positively associated with CS-discrimination at the follow-up test.

In addition, findings of clinical (analogue) studies testing the relevance of expectancy violation are also mixed. While some studies support the role of expectancy violation during exposure therapy (Guzick et al., 2020; Salkovskis et al., 2007) others report no association between expectancy violation and therapy outcome (Blakey et al., 2019; de Kleine et al., 2017; Raes et al., 2011; Scheveneels, Boddez, Van Daele, et al., 2019). Most of these studies, however, used correlational designs: Expectancy violation was measured by asking participants for their subjective ratings. While these correlational designs can be useful for detecting relationships, correlation does not imply causation – which is a prerequisite to interpret these relationships mechanistically. To demonstrate its impact on extinction learning, it is thus necessary to manipulate expectancy violation systematically. Therefore, the goal of the current study is to experimentally test the influence of expectancy violation on extinction retention. Specifically, we expected that increased expectancy violation during fear extinction leads to a) lower threat ratings towards the conditioned stimulus directly after extinction, and lower threat ratings and lower US-expectancy b) at a spontaneous recovery test as well as c) at a reinstatement test on the day following fear extinction.

We used a web-based fear conditioning protocol in which participants are divided into two groups. During extinction, the high expectancy violation (HE) group sees only the states of the CS that are strongly associated with an US. Thus, a strong expectancy violation is possible. In contrast, the low expectancy violation (LE) group is presented only with the CS states that are weakly related to the US. Therefore, the magnitude of expectancy violation is minimized. Furthermore, in the current study, we exploit the benefits of conducting a fear conditioning paradigm remotely. Recent evidence suggests that fear conditioning data can be economically collected outside of the laboratory context (McGregor et al., 2021; Purves et al., 2019; Stegmann et al., 2021; Wise & Dolan, 2020), providing a unique opportunity to draw on a larger and more diverse participant pool.

## Method

All hypotheses and methods of this study were preregistered at https://osf.io/7bgtv

### Subjects

In total, 127 individuals completed the web-based paradigm. Participants had to be at least 18 years and were excluded if they were classified as non-learners (i.e., if they reported higher US-expectancy ratings for the least reinforced conditioned size compared to the most reinforced conditioned size; *n* = 22) or if they admitted to having muted their computer audio during the main task (*n* = 1) or rated the volume of the US with zero (i.e., total silence, *n* = 3). After exclusion, complete datasets of 101 participants (77 females) with a mean age of 21.8  ±  4.3 years remained for analyses. All experimental procedures were approved by the ethics committee of the Department of Psychology at the University of Würzburg. Procedures were in agreement with the Declaration of Helsinki. All participants provided informed consent online. They received either course credits or could join a lottery for one of five 50€ coupons as compensation.

### Stimuli and Materials

The CS consisted of a light grey sphere, which was centrally presented on a dark grey background. To manipulate threat imminence, the size of the CS varied between the baseline size of either 1.25% or 26.25% and eight potential final sizes (5%, 7.5%, 10%, 12.5%, 15%, 17.5%, 20%, and 22.5%) relative to the participant’s screen size. The stimulus size in- or decreased from the baseline to the final size, resulting in a visual 3D effect of an approaching/receding sphere. To enhance this effect, two circular lines with a radius of 15% and 22.5% were displayed.

**Figure 2:**
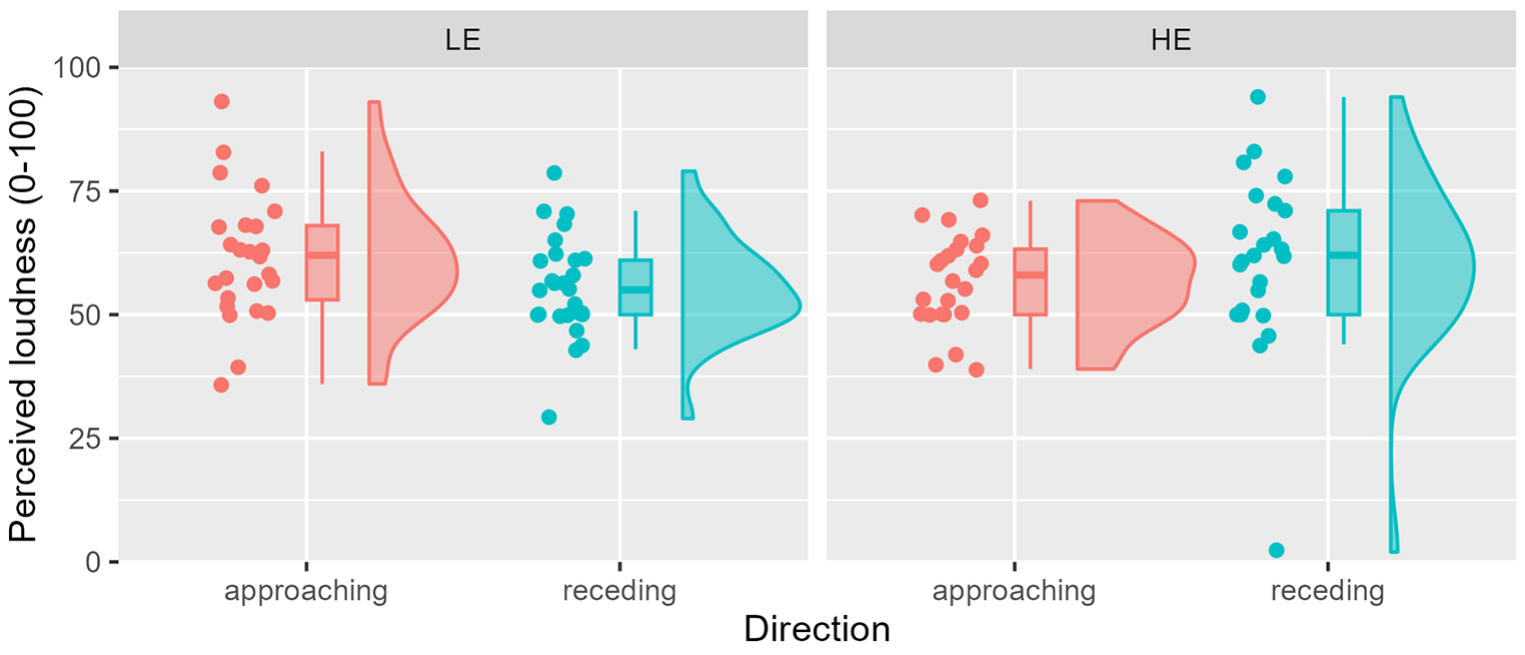
Rain Cloud Plot of the Perceived Volume of the US Asked at the End of Day 2 *Note.* Code based on Allen et al. (2021). It should be noted that one participant in the HE group gave a loudness rating of 2. In order to avoid arbitrary post-hoc cut-offs, we decided not to exclude this outlier from the analyses. However, in exploratory re-analyses, excluding this participant did not change our results.

The US was a female scream with a duration of 2.5 s (MaderaDelEste Films, 2011). At the beginning of the experiment, participants had to adjust the volume of their computer using a pleasant example melody (Frei, 2020) so that it was perceived as 5 on a scale from 0 (absolute silence) to 10 (unbearable volume). The setting was to be maintained during the experiment. After the main experiment, participants were asked to rate the loudness of the scream using the same scale. There was no difference in perceived loudness among groups, *F*(3, 97) = 1.26, *p* = .292 (see Figure 2).

### Design and Procedure

**Figure 3:**
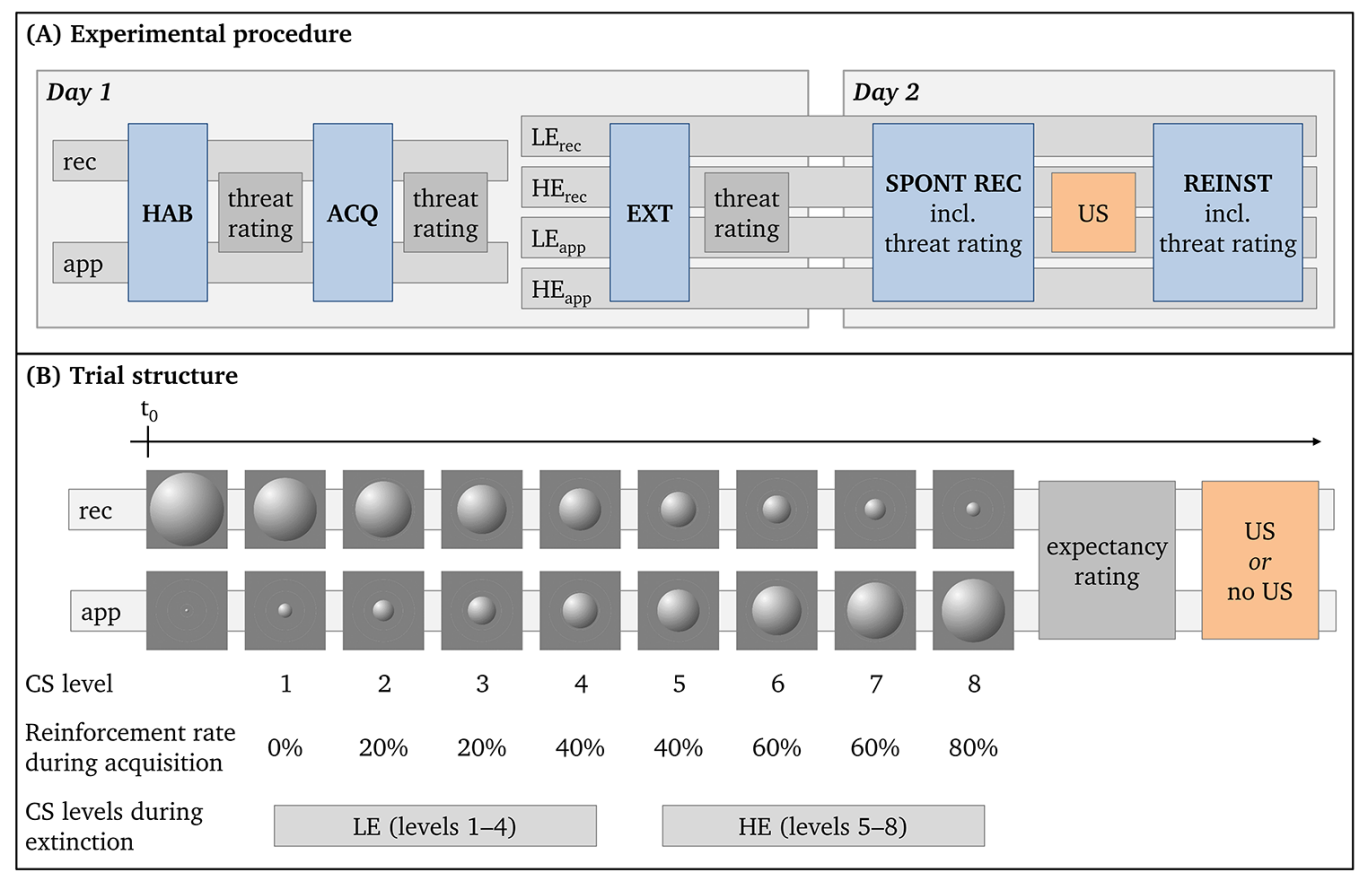
(A) Summary of the Experimental Procedure and (B) Description of the Trial Structure *Note.* (A) On Day 1, participants were divided into the receding (rec) and approaching (app) CS groups, before undergoing a habituation (HAB) and fear acquisition phase (ACQ). In the subsequent extinction phase (EXT), participants were again divided into two groups. To experimentally manipulate the extent of expectancy violation, one group (low expectancy violation; LE group) was presented only with the CS levels associated with low US likelihoods (CS Levels 1 – 4), whereas the other group (high expectancy violation; HE group) saw only the CS levels associated with high US likelihoods (CS Levels 5 – 8). On Day 2, all participants completed a spontaneous recovery (SPONT REC) and reinstatement (REINST) test. Threat ratings were collected for each CS level after each phase on Day 1. On Day 2, threat ratings for each CS level in each phase were collected directly after the expectancy rating for the respective CS level. (B) Each trial started with the presentation of the baseline-sized CS (smallest size for the approaching groups or largest size for the receding groups). After 0.8 – 1.3 s, the CS started to become larger/smaller until it reached one of the 8 final sizes. Once reaching its final size, participants were asked to rate the likelihood of being presented with an US (US expectancy rating). During acquisition, US were then presented according to the specific reinforcement rate related to the CS level before the CS returned to its baseline size. Note, that no US expectancy ratings were collected during habituation. In the habituation, spontaneous recovery, and reinstatement phases, the CS reached each final size once, while in acquisition it reached each final size five times. In extinction, each of the group's four final sizes were reached ten times.

*Day 1:* After giving informed consent, participants completed German versions of a demographic questionnaire and the Anxiety Sensitivity Index-3 (ASI-3; Kemper et al., 2009; Taylor et al., 2007), using an online survey platform (www.formr.org, Arslan et al., 2020). They were then redirected to www.pavlovia.org, where the main experiment took place (Peirce, 2007). The conditioning protocol on Day 1 consisted of a habituation, acquisition, and extinction phase (see Figure 3). During habituation, each CS level was presented once. Each trial started with the presentation of the baseline-sized CS (either 1.25% or 26.25% relative to the participant’s screen). After 0.8 – 1.3 s, the CS started to become larger/smaller (with a median rate of 6.8% per s) until it reached one of the 8 final sizes (5%, 7.5%, 10%, 12.5%, 15%, 17.5%, 20%, and 22.5%). Once reaching its final size, the CS returned to its baseline size with the same velocity. Since we expected that larger, approaching stimuli are perceived as inherently more threatening (Coker-Appiah et al., 2013), the CS for one half of the participants started at its smallest size and became larger (baseline size: 1.25%; CS level 1: 5% – CS level 8: 22.5%; approaching CS group), whereas the CS for the other half started at its largest size and became smaller (baseline size: 26.25%; CS level 1: 22.5% – CS level 8: 5%; receding CS group).

During acquisition, each CS level was presented five times (40 total trials) in a randomized order with the following conditions: no CS level should be presented three times in a row and the US should not be presented three times in a row. In each trial, when the stimulus had reached its final size, participants were asked to rate how much they expected the US on a visual analog scale from 0 ("very unlikely") to 100 ("very likely"). Subsequently, the US were presented according to the following pattern: no US were presented at CS level 1 (0% reinforcement rate; RR), one US was presented at CS Levels 2 and 3 (20% RR), two US were presented at CS Levels 4 and 5 (40% RR), three US were presented at CS Levels 6 and 7 (60% RR), and four US were presented at CS level 8 (80% RR). The trial ended with the CS returning to its baseline size.

In the subsequent extinction phase, participants were again divided into two groups. To experimentally manipulate the extent of expectancy violation, one group (low expectancy violation; LE group) was presented only with the CS levels associated with low US likelihoods (CS Levels 1 – 4), whereas the other group (high expectancy violation; HE group) saw only the CS levels associated with high US likelihoods (CS Levels 5 – 8). Each respective CS level was presented 10 times (40 trials in total). Importantly, no US was administered during the extinction phase and participants received no instruction about the CS-US contingencies.

*Day 2:* In the morning of the following day, participants received an email containing the hyperlink for the second part of the main experiment, consisting of spontaneous recovery and reinstatement test. At the beginning, participants were asked to re-adjust the volume of their computer. To test for spontaneous recovery, each CS level was presented once while online US-expectancy ratings were collected as described above. For the subsequent reinstatement test, a single US was delivered before each CS level was presented again.

In addition to the online US-expectancy ratings, participants were asked to rate the perceived threat (*“*How threatening do you perceive this stimulus?*”*) for each CS level on a visual analogue scale from 0 (“very harmless”) to 100 (“very threatening”) after each phase (i.e., habituation, acquisition, extinction) and for spontaneous recovery and reinstatement.

### Statistical Analysis

All statistical analyses were conducted with R 4.1.2 (R Development Core Team, 2021). The afex package (Singmann et al., 2020) was used for ANOVA with type 3 sum of squares, the effectsize package (Ben-Shachar et al., 2020) was used to calculate omega squared (ω^2^), and the emmeans package (Lenth, 2023) was used for simple contrasts. For acquisition, spontaneous recovery, and reinstatement, mean differences in threat and US-expectancy ratings were analyzed separately using 2 (expectancy violation: HE vs LE; between-subject factor) x 2 (CS direction: approaching vs receding; between-subject factor) x 8 (CS level: CS Levels 1 – 8; within-subject factor) mixed ANOVAs. Threat ratings after habituation were analyzed using the identical procedure. Significant main and interaction effects were followed-up with simple contrasts. To quantify the extent of expectancy violation, US-expectancy ratings obtained during the extinction phase were summarized analogous to Scheveneels, Boddez, Vervliet, et al. (2019) and compared between groups using a 2 (expectancy violation: HE vs LE) x 2 (CS direction: approaching vs receding) ANOVA. Since the true probability of an US-occurrence during extinction was always zero, expectancy violation can be calculated as the trial-wise US-expectancy ratings minus zero. Thus, the sum of the US-expectancy ratings across individual trials yields the total value of expectancy violation. A significance level of .05 was used for all analyses and Greenhouse–Geisser correction was applied where appropriate (Greenhouse & Geisser, 1959). Throughout this manuscript, we report corrected degrees of freedom, corrected *p* values and the omega squared (ω^2^). Data and code for the reported analyses are available at https://osf.io/tg2fb/.

## Results

### Online Expectancy Ratings

**Figure 4:**
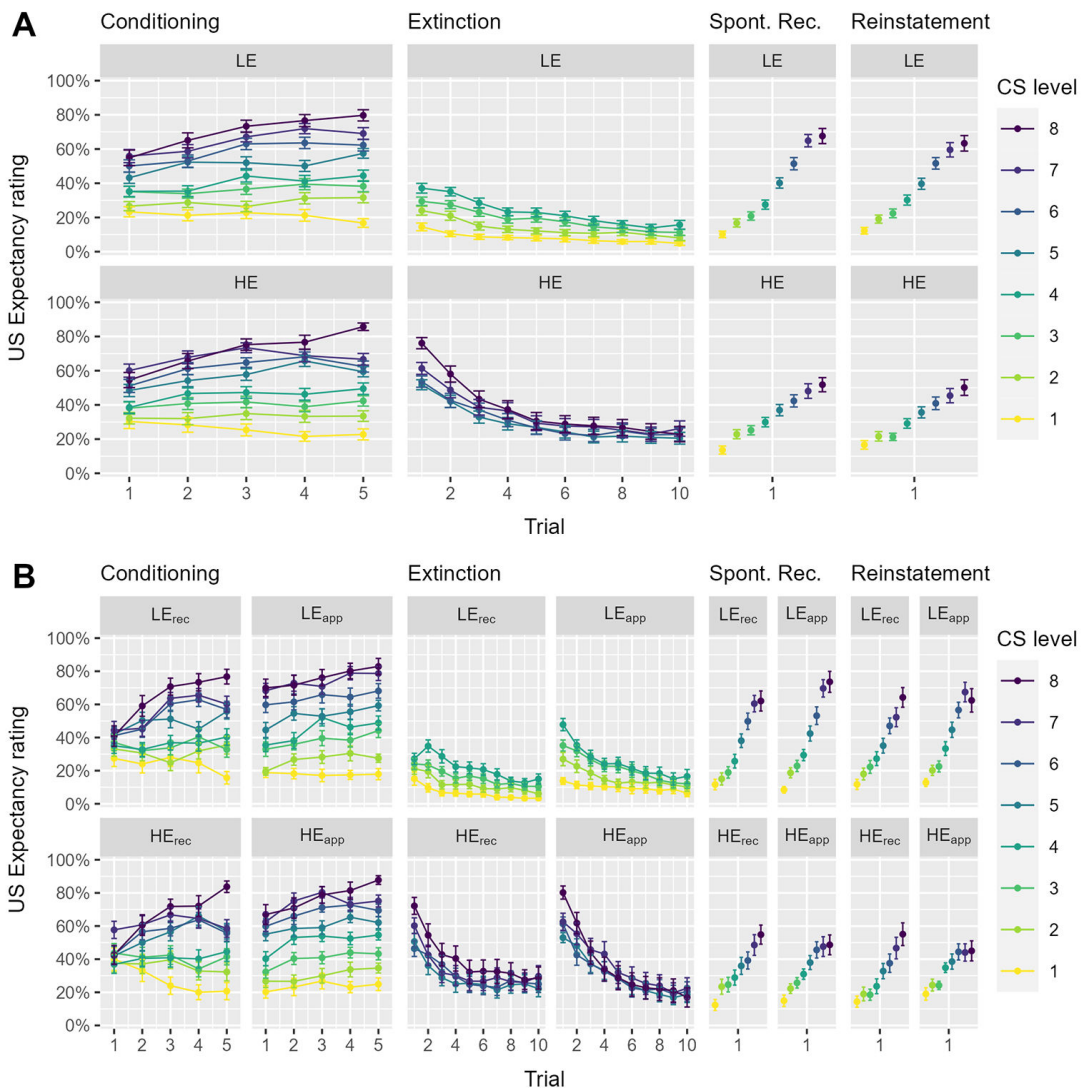
US-Expectancy Ratings *Note.* (A) Summary of the US-expectancy ratings on single trial level for low (LE) and high (HE) expectancy violation groups, and each experimental phase (error bars indicate the standard error of the mean). (B) Shows the same results separately for the approaching (app) and receding (rec) CS groups. Conditioned stimulus level (CS level) corresponds to threat imminence, i.e., larger physical sizes for approaching CS groups and smaller physical sizes for receding CS groups.

All results for US-expectancy ratings are illustrated in Figure 4. The analysis of the last presentation of each stimulus in the acquisition phase demonstrated successful fear conditioning as indexed by a significant main effect of CS level, *F*(5.73, 555.56) = 112.90, *p* < .001, ω^2^ = .44, indicating that participants expected the US more strongly with increasing threat imminence (larger physical sizes in the approaching CS groups, smaller physical sizes in the receding CS groups). In addition, there was a main effect of CS direction, *F*(1, 97) = 8.10, *p* = .005, ω^2^ = .07, which was further qualified by a significant interaction between CS level and CS direction, *F*(5.73, 555.56) = 2.57, *p* = .020, ω^2^ = .01. Together, these results indicate higher US-expectancy ratings in the approaching compared to the receding CS groups, particularly, for the 6^th^, *t*(97) = 2.72, *p* = .008, and 7^th^ level, *t*(97) = 3.84, *p* < .001, of CS level (all other levels, *p*’s > .050), suggesting that physical size interfered with acquisition learning, i.e., that larger physical sizes of an approaching CS are more readily associated with the occurrence of the US than smaller physical CS levels in the receding group. Importantly, there were no differences between HE and LE groups, *p*’s > .259.

During extinction training, the HE group showed higher summarized US-expectancy ratings and thus stronger expectancy violation than the LE group, *F*(1, 97) = 25.08, *p* < .001, ω^2^ = .19, implying a successful experimental manipulation of expectancy violation.

On Day 2 at the spontaneous recovery test, there was a main effect of CS level, *F*(2.97, 287.72) = 96.82, *p* < .001, ω^2^ = .35, demonstrating higher expectancy ratings with increasing threat imminence in all groups, while a significant CS level x expectancy violation interaction, *F*(2.97, 287.72) = 6.73, *p* < .001, ω^2^ = .03, indicates higher US-expectancy ratings and thus a stronger recovery of conditioned fear for LE compared to HE groups at the 7^th^: *t*(97) = 3.03, *p* = .003, and 8^th^: *t*(97) = 2.66, *p* = .009, CS levels (all other levels, *p*’s > .078). No effect of direction reached significance, *p*’s > .366.

The US presentation at reinstatement did not substantially change these results. The main effect of CS level, *F*(3.08, 298.44) = 76.64, *p* < .001, ω^2^ = .29, and the CS level x expectancy violation interaction, *F*(3.08, 298.44) = 4.05, *p* = .007, ω^2^ = .02, remained significant. Again, LE compared to HE groups reported higher expectancy ratings at the 6^th^: *t*(97) = 2.17, *p* = .032, 7^th^: *t*(97) = 2.47, *p* = .015, and 8^th^: *t*(97) = 2.05, *p* = .044, CS levels (all other levels, *p*’s > .167). No effect of direction reached significance, *p*’s > .161.

### Threat Ratings

**Figure 5:**
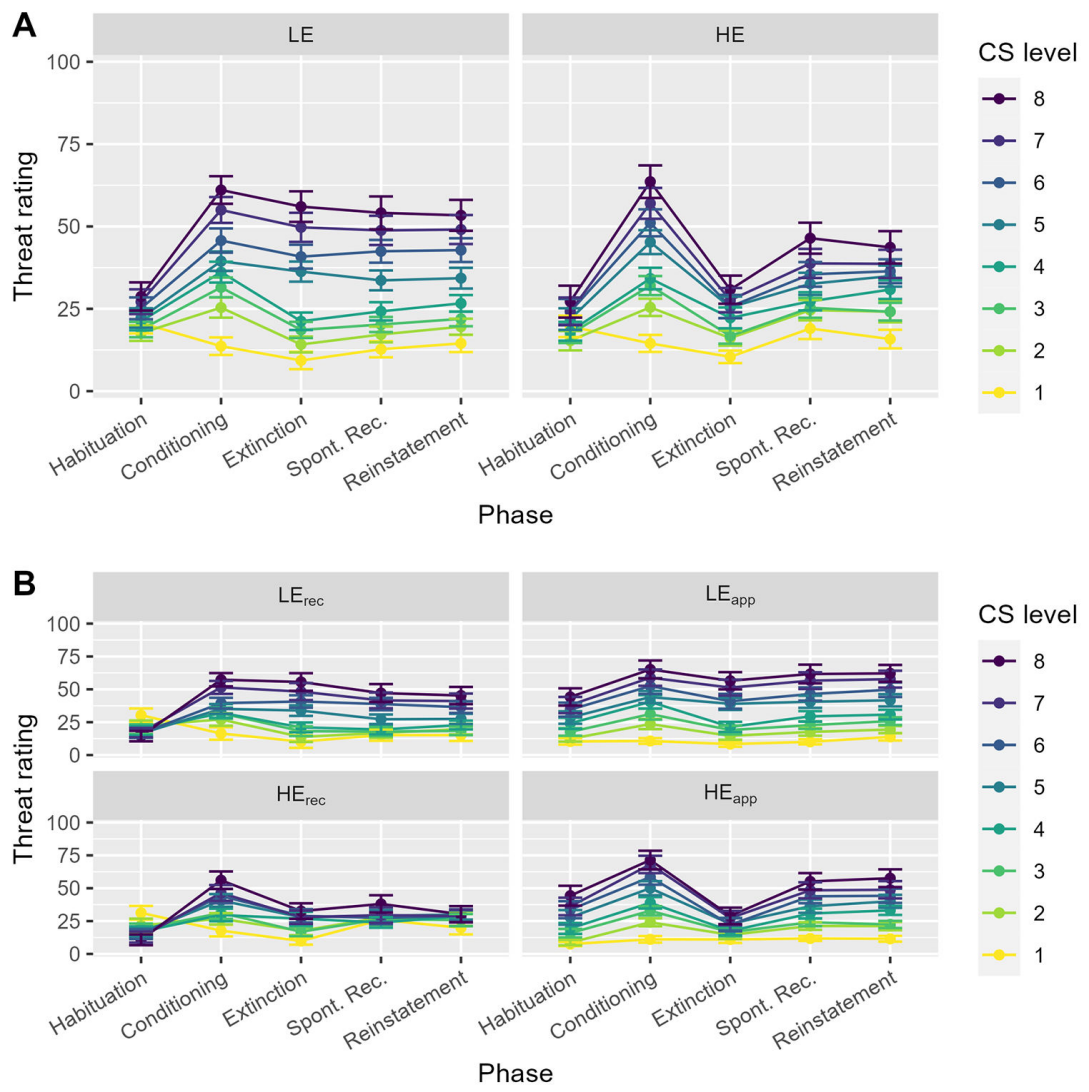
Threat Ratings *Note.* (A) Summary of the threat ratings for low (LE) and high (HE) expectancy violation groups, and each experimental phase (error bars indicate the standard error of the mean). (B) Shows the same results separately for the approaching (app) and receding (rec) CS groups. Conditioned stimulus level (CS level) corresponds to threat imminence, i.e., larger physical sizes for approaching CS groups and smaller physical sizes for receding CS groups.

After habituation, the 2x2x8 ANOVA for subjective threat ratings revealed a significant main effect of CS level, *F*(2.08, 201.70) = 10.10, *p* < .001, ω^2^ = .03. Crucially, there was also a significant interaction between CS level and CS direction, *F*(2.08, 201.70) = 46.15, *p* < .001, ω^2^ = .12, indicating higher threat ratings for increasing CS levels (i.e., increasing sizes) in the approaching CS groups and higher threat ratings for decreasing CS levels (i.e., increasing sizes) in the receding CS groups (see Figure 5).

 This result is in line with the notion that visual stimuli appear inherently more threatening with increasing physical size, i.e., lower CS levels in receding CS groups and higher CS levels in approaching CS groups.

At the end of the acquisition phase, successful conditioning was indexed by a significant main effect of CS level, *F*(2.91, 282.23) = 92.07, *p* < .001, ω^2^ = .27. In addition, there was a CS direction x CS level interaction, *F*(2.91, 282.23) = 5.58, *p* = .001, ω^2^ = .02. Taken together, these results demonstrate that participants perceived more threat with increasing threat imminence. Yet, physical size of the CS still influenced threat ratings as indexed by slightly higher threat ratings in the approaching CS compared to the receding CS groups at the 4^th^: *t*(97) = 1.95, *p* = .055, 5^th^: *t*(97) = 1.98, *p* = .050, 6^th^: *t*(97) = 2.56, *p* = .012, 7^th^: *t*(97) = 2.45, *p* = .016, and 8^th^: *t*(97) = 1.81, *p* = .074, CS level. Please note, that the 8^th^ CS level was the largest physical size in the approaching CS group but the smallest physical size in the receding CS group. Importantly, no differences between LE and HE groups were found, *p*’s > .610.

Directly after extinction, the effect of the expectancy violation manipulation was evident in a significant CS level x expectancy violation interaction, *F*(2.53, 245.24) = 12.42, *p* < .001, ω^2^ = .04, which could be retrieved in addition to main effects of expectancy violation, *F*(1, 97) = 6.18, *p* = .015,, ω^2^ = .05, and CS level, *F*(2.53, 245.24) = 58.70, *p* < .001, ω^2^ = .19. As illustrated in Figure 5, the HE groups reported lower threat ratings compared to the LE groups at the 5^th^: *t*(97) = 2.45, *p* = .016, 6^th^: *t*(97) = 2.98, *p* = .004, 7^th^: *t*(97) = 3.78, *p* < .001, and 8^th^: *t*(97) = 3.95, *p* < .001, CS level, while there were no differences for smaller CS levels, *p*’s > .579. Furthermore, we found no effect of CS direction, *p*’s > .521. To further analyze the effect of expectancy violation on threat ratings, we tested the differences between groups from acquisition to extinction. Indeed, for the HE group, we found a decrease in threat ratings for all CS levels, *p*’s < .003, except for the lowest level, *t*(48) = 1.62, *p* = .112, while threat ratings in the LE groups decreased only for the four lowest (CS Levels 1 – 4), *p*’s < .015, but not for the four highest levels (CS Levels 5 – 8), *p*’s > .184, suggesting that participants in the LE groups still perceived higher CS levels as threatening.

For threat ratings at spontaneous recovery on Day 2, the main effect of CS level, *F*(2.36, 229.23) = 54.61, *p* < .001, ω^2^ = .18, and the interaction between CS level and expectancy violation, *F*(2.36, 229.23) = 4.38, *p* = .009, ω^2^ = .01, remained significant. Yet, simple contrasts revealed no significant differences between LE and HE groups at the individual CS levels, all *p*’s > .063. In addition, there was a CS level x CS direction interaction, *F*(2.36, 229.23) = 8.10, *p* < .001, ω^2^ = .03, indicating spontaneous recovery of the effect of physical size on threat ratings similar to the results of the habituation phase. Together, these results suggest that the differential effect of expectancy violation on threat ratings did not persist until the second day of the study. To substantiate this finding, we also analyzed change scores between the end of acquisition and spontaneous recovery at the individual CS levels separately for the HE and LE groups. Student’s *t*-tests revealed decreased threat ratings for CS Levels 3 to 8 in the HE groups, *p*’s < .029, and decreased threat ratings for CS Levels 2 to 5 in the LE groups, *p*’s < .018.

A similar pattern of results could be obtained for threat ratings at the reinstatement test. Main effects of CS direction, *F*(1, 97) = 5.31, *p* = .023, ω^2^ = .04, and CS level, *F*(2.08, 201.73) = 54.39, *p* < .001, ω^2^ = .18, were qualified by significant interactions between CS direction and CS level, *F*(2.08, 201.73) = 9.54, *p* < .001, ω^2^ = .03, as well as between CS level and expectancy violation, *F*(2.08, 201.73) = 3.68, *p* = .025, ω^2^ = .01. Higher CS levels were generally associated with higher threat ratings, while physical size interfered with actual threat imminence similarly to the description above. Again, simple contrasts revealed no significant differences between LE and HE groups at the individual CS levels, *p*’s > .079.

## Discussion

The main goal of our study was to provide causal evidence for the influence of expectancy violation on extinction retention. To this end, we employed a web-based fear conditioning protocol, in which we manipulated the magnitude of expectancy violation during the extinction learning phase. Subjective threat and US-expectancy ratings were obtained throughout the acquisition and extinction phase on Day 1, as well as during a spontaneous recovery and reinstatement test on Day 2.

In line with previous fear conditioning studies, our results showed successful fear acquisition and extinction for US-expectancy and threat ratings, indicating that participants learned the CS-US and CS-noUS associations. Consistent with our manipulation of expectancy violation, however, the HE groups reported higher expectancy ratings than the LE groups. Because no US was presented during extinction, higher US-expectancy ratings also imply stronger expectancy violation, and according to the inhibitory learning model, stronger expectancy violation should have led to a stronger formation of the CS-noUS association (Craske et al., 2014; Craske et al., 2022; Scheveneels, Boddez, Vervliet, et al., 2019). As predicted by the inhibitory learning model, the HE groups indeed reported lower subjective threat compared to the LE groups at the end of the extinction phase on Day 1, providing causal evidence for the notion that the strength of expectancy violation is related to the decline of subjective threat during fear extinction.

On the second day, results for US-expectancy and threat ratings during the spontaneous recovery and reinstatement test were less conclusive. Whereas reduced expectancy ratings, and thus, a stronger retrieval of the CS-noUS association could be retrieved for the HE compared to LE groups, we found no substantial group differences for threat ratings. These findings indicate that the strength of expectancy violation did influence the extent of extinction retention, however, the effect was not as large as would have been expected according to the inhibitory learning model. This small effect might be due to extinction learning took place directly after fear acquisition and, therefore, might be influenced by the immediate extinction deficit. The immediate extinction deficit refers to the phenomenon that extinction retrieval is impaired for shorter intervals compared to longer intervals (e.g., 24 hours) between initial fear acquisition and subsequent extinction training and has been previously demonstrated in rodent and human studies (Chang et al., 2010; Huff et al., 2009; Maren, 2014; Merz et al., 2016). However, it is important to mention that on Day 2 we could retrieve the expected results for US-expectancy ratings, i.e., reduced US-expectancy ratings and thus a stronger retrieval of the CS-noUS association for the high compared to low expectancy violation groups, as predicted by the inhibitory learning model. Yet, the CS-noUS association did not appear to inhibit the perceived threat. Recently, it has been suggested that US-expectancy ratings are more likely to represent the conscious, cognitive component (Boddez et al., 2013), whereas threat ratings are more likely to capture the affective component of the fear response (Constantinou et al., 2021; Lonsdorf et al., 2017). Taken together, our results suggest that expectancy violation plays an important role in fear extinction, but it is unclear how it translates to changes in the affective component of the fear response.

Crucially, this finding is consistent with experience from clinical psychology and previous empirical findings. Patients with anxiety disorders usually know that their fears are irrational and are aware that the probability of their feared event occurring is low (Zimmerman et al., 2010). Yet, they report intense affective reactions. In a similar line of thought, Buchholz et al. (2022) compared treatment outcomes after exposure therapy following cognitive restructuring and vice versa. According to the inhibitory learning theory cognitive restructuring prior to exposure exercises should reduce threat expectancies and thus hinder expectancy violation. Indeed, patients who received cognitive restructuring before exposure showed a trend toward reduced expectancy ratings. However, contrary to the predictions of the inhibitory learning theory, the cognitive intervention did not attenuate the magnitude of change of expectancies due to exposure. In addition, the treatment outcomes of both groups were similar after treatment and at follow-up. In an analogous fear conditioning paradigm, Scheveneels, Boddez, De Ceulaer, et al. (2019) instructed half of the participants before extinction that the probability of the US will be small, whereas the control group did not receive this information. According to the inhibitory learning theory, this safety information should attenuate inhibitory learning and thus lead to an increased return of fear. Although participants in the informed group had a less pronounced decrease in US expectancies during extinction (which is consistent with the assumptions of the inhibitory learning model), it did not promote return of fear. On the contrary, the safety information reduced the return of fear compared to the control group. Combined with the results of our current study, these findings underscore that the violation of conscious expectancies does not directly translate to the outcome of exposure therapy. In line with this, a recent therapy study (Pittig et al., 2023) showed that not expectancy violation per se but rather how patients changed their threat expectancies after exposure exercises, calculated as pre-minus-post-exposure expectancy, i.e., “Imagine repeating the same exposure practice. How likely is it that the aversive outcome will occur this time?” (Craske et al., 2022), predicted treatment outcome.

There are also some limitations that need to be discussed in the context of the current study. First, we found strong effects of CS direction. As expected, threat ratings after habituation revealed that CS physical size was associated with higher threat ratings, such that closer CS appeared generally more threatening. In line with preparedness theories of fear learning (Coker-Appiah et al., 2013; Mineka & Öhman, 2002; Öhman & Mineka, 2001), we also found that the CS direction interfered with fear conditioning, i.e., larger physical CS sizes were more readily associated with the occurrence of the US than smaller sizes during fear acquisition. Importantly, the effect of CS direction on US-expectancy and threat ratings diminished during extinction learning. However, we found a strong return of this inherent fear in threat ratings during the spontaneous recovery and reinstatement test, suggesting that despite participants in the receding groups had learned that larger physical sizes indicated relative safety, they almost reverted to pre-acquisition threat levels, paralleling the difficulties in treating pathological forms of fear, as most anxiety disorders are rooted in evolutionarily prepared fears (e.g., fear of heights, spiders, snakes).

It is also important to mention that this study was conducted remotely only, and therefore, we were not able to record physiological measures of the fear response. Even though ratings are a valid and important measure of subjective threat perception (Boddez et al., 2013), future studies should seek complementary evidence from physiological indices of defense system activation, such as cardiovascular or electrodermal activity (Ojala & Bach, 2020). In contrast to laboratory studies, we were not able to standardize US-intensities and had to rely on participants’ self-reported perceived loudness, which was collected at the end of Day 2. Based on these ratings and in combination with the US-expectancy ratings, we excluded participants who turned off their volume. Nevertheless, the average US-intensity could be lower than in laboratory studies, and replications with offline samples are needed to ensure that effects remain consistent across different methods of stimulus delivery. Importantly, when using a human scream as US, successful fear conditioning was already reported at US-intensities below 80 dB (Beaurenaut et al., 2020).

In summary, the present web-based fear conditioning study demonstrated that experimentally increasing the magnitude of expectancy violation increased extinction retention for US-expectancy ratings, but this did not affect subjective threat ratings on Day 2. Future studies need to further test the predictions of the inhibitory learning model, particularly how violation of conscious expectancies may translate to subjective feelings and symptoms of anxiety. This study provided a paradigm to experimentally target these processes.

## Supplementary Materials

The Supplementary Materials contain the following items (for access see Index of Supplementary Materials below):

- Pre-registration protocol for all hypotheses and methods of the study
- Data and code for the analyses reported in this article

GromerD.
HildebrandtL. K.
StegmannY.
 (2021). Supplementary materials to "The role of expectancy violation in extinction learning: A two-day online fear conditioning study"
[Pre-registration protocol]. PsychOpen. https://osf.io/7bgtv
10.32872/cpe.9627PMC1050825837732150

GromerD.
HildebrandtL. K.
StegmannY.
 (2023). Supplementary materials to "The role of expectancy violation in extinction learning: A two-day online fear conditioning study"
[Research data and code]. PsychOpen. https://osf.io/tg2fb/
10.32872/cpe.9627PMC1050825837732150

## Data Availability

Data and code for the analyses reported in this article are freely available (Gromer, Hildebrandt, & Stegmann, 2023)
